# Gestational Diabetes Mellitus in Singleton and Twin Pregnancies: A Comparison of Fetomaternal Outcomes

**DOI:** 10.3390/diagnostics16040632

**Published:** 2026-02-22

**Authors:** Selina Balke, Izabela A. Kotzott, Annette Aigner, Petra Weid, Wolfgang Henrich, Joachim W. Dudenhausen, Josefine T. Königbauer

**Affiliations:** 1 Charité—Universitätsmedizin Berlin, Corporate Member of Freie Universität Berlin and Humboldt-Universität zu Berlin, Klinik für Geburtsmedizin, Charitéplatz 1, 10117 Berlin, Germany; 2 Charité—Universitätsmedizin Berlin, Corporate Member of Freie Universität Berlin and Humboldt-Universität zu Berlin, Klinik für Gynäkologie mit Brustzentrum, Charitéplatz 1, 10117 Berlin, Germany; 3 Charité—Universitätsmedizin Berlin, Corporate Member of Freie Universität Berlin and Humboldt-Universität zu Berlin, Institute of Biometry and Clinical Epidemiology, Charitéplatz 1, 10117 Berlin, Germany; 4 Charité—Universitätsmedizin Berlin, Corporate Member of Freie Universität Berlin and Humboldt-Universität zu Berlin, Center for Stroke Research Berlin, Charitéplatz 1, 10117 Berlin, Germany

**Keywords:** gestational diabetes, GDM, twin, pregnancy, insulin, OGTT, fetal growth

## Abstract

**Background:** Gestational diabetes mellitus (GDM) complicates a significant number of pregnancies and is associated with both short- and long-term risks for the mother and child. Twin pregnancies are inherently high risk, and the coexistence of GDM may amplify these risks. While the effects of GDM in singleton pregnancies have been widely studied, data on its impact in twin gestations remain limited. The aim of this study was to determine differences regarding metabolic characteristics, treatment requirements, and maternal as well as fetal outcomes between twin and singleton pregnancies with GDM to contribute to improved perinatal care. **Methods:** This retrospective study included obstetric data from 73 twin pregnancies (146 neonates) and 1664 singleton pregnancies with a GDM diagnosis at a tertiary perinatal center in Berlin, Germany, between 2015 and 2022. Baseline characteristics and perinatal outcomes were assessed. Adjusted multiple linear and logistic regression analyses were used for group comparisons. **Results:** Women with GDM in twin and singleton pregnancies exhibited comparable glucose values in the 75 g oral glucose tolerance test (OGTT) (median fasting: 95 vs. 96 mg/dL; 1 h: 183 vs. 183 mg/dL; 2 h: 144 vs. 139 mg/dL). Despite this, insulin therapy was required significantly less often in twin (5.5%) compared to singleton pregnancies (22.3%) (OR = 0.86; 95% CI: 0.78–0.96). Among insulin-treated women, combined insulin therapy was most common in twins (75%), while singleton mothers most frequently received long-acting insulin alone (61.7%), followed by combined therapy (31.3%) and short-acting insulin alone (7%). Birthweight was significantly lower in twins (β = –0.83 kg; 95% CI: –0.98 to –0.69), and when evaluated using twin-based growth standards, twins were more likely to be classified as having intrauterine growth restriction (IUGR, <3rd percentile) (OR = 3.37; 95% CI: 0.96–9.11), being small for gestational age (SGA, <10th percentile) (OR = 2.50; 95% CI: 1.23–4.76), or having a birthweight below the 30th percentile (OR = 6.11; 95% CI: 3.49–11.12). No large-for-gestational-age (LGA, >90th percentile) neonates were observed in the twin group. **Conclusions:** GDM manifests differently in twin and singleton pregnancies. Despite similar OGTT values, twin mothers require insulin less frequently. Growth-related complications such as IUGR and SGA are significantly more frequent in twins, likely reflecting the physiological constraints of multiple gestations rather than GDM itself. Conversely, LGA is predominantly a concern in singleton pregnancies. These findings underscore the need for individualized diagnostic criteria and management strategies for GDM in twin pregnancies.

## 1. Introduction

Gestational diabetes mellitus (GDM) is a common pregnancy complication, defined as glucose intolerance diagnosed during pregnancy—most often arising in the second or third trimester [1]. In Germany, pregnant women are offered a two-step screening for GDM between the 24th and 28th week of pregnancy. The first step involves a 50 g glucose challenge test, and if the result is abnormal (≥135 mg/dL after one hour), a 75 g OGTT is performed on another day. GDM is diagnosed if any of the following thresholds are exceeded: fasting ≥ 92 mg/dL, 1 h ≥ 180 mg/dL, or 2 h ≥ 153 mg/dL [2]. The 75 g OGTT may also be used as a primary screening tool in women with relevant risk factors for GDM.

GDM is associated with a range of adverse outcomes for both mother and child, including an elevated risk of preeclampsia, cesarean delivery, and large-for-gestational-age (LGA) neonates [3,4,5]. In Germany, GDM affected approximately 8.5% of pregnancies in 2021, and its prevalence has been steadily increasing [6]. This rise is likely attributable to the growing prevalence of known risk factors such as maternal obesity and advanced maternal age [7,8,9,10].

While most research to date has focused on singleton pregnancies, demographic changes and advances in reproductive medicine have led to a growing number of twin pregnancies, which merit closer examination with regard to GDM [11,12,13]. Twin gestations are themselves recognized as a risk factor for the development of GDM, with multiple studies reporting a significantly higher incidence of GDM among women pregnant with twins compared to singletons [14,15,16]. Furthermore, twin pregnancies are more frequently associated with obstetric complications such as preterm birth, gestational hypertension, preeclampsia, and fetal growth abnormalities, including intrauterine growth restriction (IUGR) and small-for-gestational-age (SGA) infants [17,18,19]. These elevated risks underscore the importance of guideline-based intensified monitoring and individualized care in twin pregnancies, in line with the Arbeitsgemeinschaft der Wissenschaftlichen Medizinischen Fachgesellschaften (AWMF) [20].

However, existing literature on GDM in twin pregnancies has yielded conflicting results. Some studies have indicated a higher risk of adverse maternal and neonatal outcomes in twin pregnancies with GDM, including increased rates of preeclampsia, cesarean section (CS), preterm birth, neonatal hypoglycemia, and SGA infants [21,22]. Conversely, other investigations suggest a potentially attenuated impact of GDM in twin pregnancies, reporting lower relative risks of cesarean delivery, neonatal intensive care unit (NICU) admission, stillbirth, and neonatal mortality compared to singleton pregnancies [23]. A more recent study even found that singleton and twin pregnancies may exhibit comparable overall risk profiles [24]. This heterogeneity in findings highlights a gap in current evidence and the need for further targeted research.

The present study aims to systematically compare maternal and neonatal outcomes in women with GDM carrying singleton versus twin pregnancies. Key areas of focus include maternal glucose metabolism, insulin therapy requirements, pregnancy complications, and neonatal outcomes. By elucidating potential differences, this research seeks to inform clinical decision-making and support tailored management strategies for optimizing maternal and fetal health in high-risk GDM pregnancies.

## 2. Methods

This study was granted ethical approval by the Charité—Universitätsmedizin Berlin ethics commission in 2023 (EA2/255/22). Informed consent was waived due to the retrospective nature of the study.

### 2.1. Data Collection

From 2015 to 2022, obstetric data from 3123 women with gestational diabetes, who presented at the GDM consultation clinic at Charité—Universitätsmedizin in Berlin, Germany, were retrospectively collected.

Maternal and neonatal data were extracted from obstetric data and documented prenatal visits at the GDM consultation clinic. Primary documentation was recorded using the software Viewpoint version 6 (GE Healthcare, Chicago, IL, USA).

The extracted maternal data included the mother’s age, first presentation at the GDM consultation clinic, results of the 50 g oral glucose challenge test (OGCT) and the 75 g oral glucose tolerance test (OGTT), insulin therapy, date and gestational week (GW) at delivery, mode of delivery, diagnosis of preeclampsia, and maternal height, weight, Body Mass Index (BMI), and weight gain during pregnancy. Neonatal data included fetal position (for twins), indication for cesarian section (for twins), shoulder dystocia, birthweight and percentile, umbilical cord pH levels, base excess (BE), and APGAR scores at 1, 5, and 10 min after birth. Fetal growth was assessed based on birthweight percentiles, which were calculated in Viewpoint using twin-based growth standards. Infants were classified as having intrauterine growth restriction (IUGR) if their birthweight was below the 3rd percentile, being small for gestational age (SGA) if below the 10th percentile, having low fetal weight if below the 30th percentile, and being large for gestational age (LGA) if above the 90th percentile.

### 2.2. Inclusion and Exclusion Criteria

Inclusion criteria were maternal age over 18 years, a GDM diagnosis in the current pregnancy based on the International Association of the Diabetes and Pregnancy Study Groups (IADPSG) criteria, and delivery at Charité—Universitätsmedizin Berlin [25]. IADPSG criteria apply if at least one of the following thresholds in the 75 g OGTT are exceeded: fasting ≥ 92 mg/dL, 1 h ≥ 180 mg/dL, or 2 h ≥ 153 mg/dL [25]. Mothers were excluded if they had preexisting diabetes mellitus or if their fasting 75 g OGTT results and delivery data were missing. Patients with multiple pregnancies other than twins (triplets and quadruplets) were also excluded from the analysis. Finally, 1664 mothers of singletons and 73 mothers of twins, and therefore 146 twin neonates, were included in this study.

### 2.3. Statistical Analysis

We descriptively report the median and interquartile ranges (IQRs) for continuous variables and absolute and relative frequencies for categorical variables, stratified by twin and singleton pregnancy.

To evaluate binary outcome parameters, binary logistic regression analyses were performed and results presented as odds ratios (OR) with corresponding 95% confidence intervals (CI). In cases where the dependent variable represented a binary fetal outcome, it was considered positive if at least one of the twin births was recorded as such. For continuous maternal outcome parameters, linear regression models were fitted, and for continuous fetal outcomes, linear mixed-effects models with a random intercept for pregnancy were used. The corresponding regression coefficients (β) along with 95% CIs were reported. BMI, maternal age, and nulliparity were considered potential confounding factors and therefore included in all regression models. Mode of conception was also identified as a possible confounder; however, this information could not be extracted from the available medical records.

SPSS Statistics by IBM (Armonk, NY, USA) (Version 28.0.1.0), R (Version 4.3.1) and additional R packages were used for statistical analysis [26,27,28].

## 3. Results

### 3.1. Baseline Characteristics

Women with twin pregnancies presented to the GDM consultation clinic two weeks earlier than those with singleton pregnancies (median: 28 vs. 30 GW). Of the twin pregnancies, 78.9% were dichorionic diamniotic, while 21.1% were monochorionic diamniotic.

Women with singleton pregnancies were slightly younger (median: 33 vs. 34 years), displayed a higher BMI (median: 27.5 vs. 25.75 kg/m^2^), and had experienced a greater number of previous pregnancies (median: 3 vs. 2) and births (median: 1 vs. 0). While 52.7% of women with singleton pregnancies were experiencing their third pregnancy or more, this applied to only 33.8% of women expecting twins. Conversely, 66.2% of twin mothers were in their first or second pregnancy, compared to 47.3% of singleton mothers. Additionally, 50.7% of women with twin pregnancies were nulliparous, compared to 31.6% of those with singleton pregnancies (Table 1).

### 3.2. Metabolism

A greater proportion of women in the singleton group underwent a 50 g OGCT compared to the twin group (54.4% vs. 36.1%). Among those tested, mothers with twin pregnancies received the 50 g OGCT slightly earlier (median: 25 vs. 26 GW). Median blood glucose levels in the test were 154 (singleton) versus 161.5 mg/dL (twin). A pathological response to the 50 g OGCT was observed in 91.6% of singleton and 96.2% of twin pregnancies.

Women with twin pregnancies also underwent the 75 g OGTT earlier than those with singleton pregnancies (median: 26 vs. 27 GW). In 13.7% of twin pregnancies, the 75 g OGTT was conducted before GW 24 + 0, compared to 11.1% of singleton pregnancies. Fasting, 1 h, and 2 h blood glucose levels during the OGTT were similar between the groups (Table 2).

### 3.3. Maternal Outcome and Birth Mode

**Figure 1:**
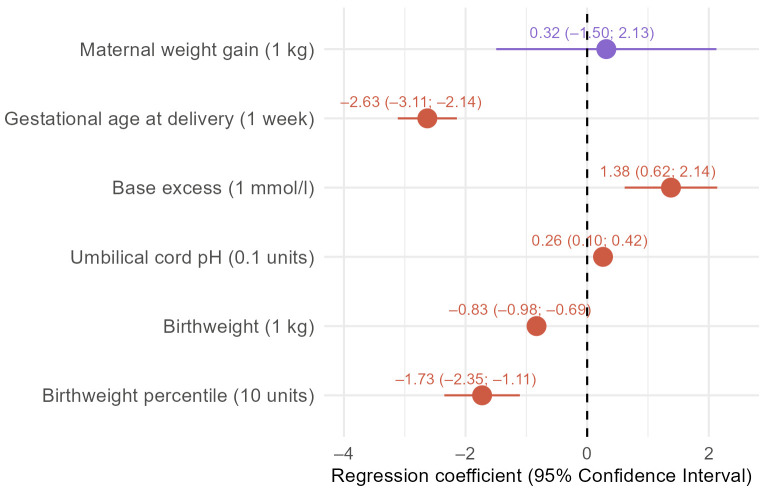
Adjusted effect of twin pregnancy on continuous fetomaternal outcomes. Results of mixed linear regression models are presented as regression coefficients (numbers out of parentheses) with corresponding 95% confidence intervals (CIs) (numbers in parentheses) for fetal and maternal outcomes, adjusted for maternal age, Body Mass Index, and parity. Maternal weight gain, base excess, and umbilical cord pH levels were slightly higher in twin pregnancies, whereas gestational age at delivery, birthweight and corresponding percentiles were lower.

Twin mothers exhibited a greater absolute (median: 12.4 vs. 11 kg) as well as relative weight gain (median: 17.3 vs. 14.9%) (Table 3). Adjusting for BMI, maternal age, and nulliparity, the maternal weight gain was similar between the groups (β = 0.32 kg [−1.50; 2.13]) (Figure 1).

**Figure 2:**
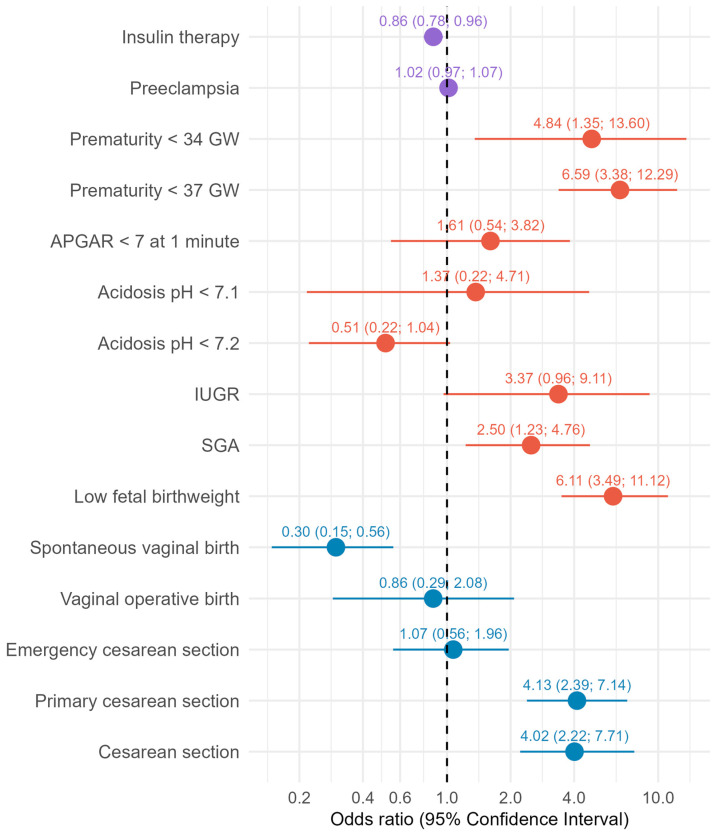
Adjusted effects of twin pregnancy on binary fetomaternal outcomes. Results from binary logistic regression models are presented as odds ratios (ORs) (numbers in parentheses) with corresponding 95% confidence intervals (CIs) (numbers out of parentheses), adjusted for maternal age, Body Mass Index (BMI), and parity. Compared to singleton pregnancies, twin pregnancies had higher odds of premature birth, cesarean section (CS), low initial APGAR score, birthweight below the 30th percentile, small-for-gestational-age (SGA) newborns, and intrauterine growth restriction (IUGR). In contrast, lower odds were observed for insulin therapy, acidosis (pH < 7.2), and both spontaneous and operative vaginal births. The odds of preeclampsia and emergency CS were comparable between twin and singleton pregnancies.

Twin pregnancies were less likely to require insulin therapy (5.5% vs. 22.3%). Long-acting insulin alone was used by 13.8% of singleton and 1.4% of twin mothers. Short-acting insulin was required alone in 1.6% of singleton and in none of the twin pregnancies. Combined insulin use was more common in singleton pregnancies (7% vs. twin: 4.1%) (Table 3). Taking into account maternal age, BMI and parity, twin mothers still had lower odds of being insulin-dependent (OR = 0.86; 95% CI: 0.78; 0.96) (Figure 2).

The proportion of vaginal deliveries was more than twice as high in the singleton group (57.7% vs. 24.6%). Spontaneous vaginal births accounted for 50.2% of deliveries in the singleton and 17.8% in the twin group. The frequency of operative vaginal births was similar between the groups (singleton: 7.5% vs. twin: 6.8%). CS was performed more frequently in twin pregnancies (75.3% vs. 42.3%). Primary CS was performed in 53.4% of twin compared to 21.8% of singleton pregnancies. In contrast, the frequency of emergency CS was comparable between the two groups (singleton: 20.6% vs. twin: 21.9%) (Table 3).

After adjustment for confounding, the odds of spontaneous vaginal birth were substantially lower among women with twin pregnancies (OR = 0.30; 95% CI: 0.15; 0.56). Conversely, twin mothers had 4-fold-higher odds of CS in general (OR = 4.02; 95% CI: 2.22; 7.71), just as of delivering via primary CS (OR = 4.13; 95% CI: 2.39; 7.14). However, the odds of emergency CS were not independently higher in twin pregnancies (OR = 1.07; 95% CI: 0.56; 1.96). The odds of vaginal operative birth were somewhat lower in twin pregnancies (OR = 0.86; 95% CI: 0.29; 2.08) (Figure 2).

Preeclampsia occurred more frequently in twin pregnancies (5.6% vs. 3.1%) (Table 3). The odds of preeclampsia were similar for both groups after adjusting for confounding (OR = 1.02; 95% CI: 0.97; 1.07) (Figure 2).

### 3.4. Fetal Position and Indications for Cesarean Section in Twin Pregnancies

At the time of delivery, 77.5% of the first-born twins were in cephalic position, while 22.5% were in breech position. The second-born twins were in cephalic position in 60.6% and in breech position in 30.9% of pregnancies. There was transverse presentation in 8.5% of second-born twins.

The most common indications for a CS in twin pregnancies were the fetal position (19.2%) and other unspecified reasons associated with the presence of a twin pregnancy, as well as maternal request (19.2%). Of all of the women, 17.3% had a repeat CS. In 15.4% of pregnancies the indications for a CS were related to fetal growth anomalies, such as (selective) intrauterine growth restriction. A further 15.4% of women were initially scheduled for a primary CS but underwent the procedure after labor began. In 3.8% of the pregnancies an emergency CS was performed to deliver the second twin after the first twin was delivered vaginally, and another 3.8% of operations were due to preeclampsia. There was one woman each with a maternal indication due to a severe chronic condition, disorder of placental implantation and unsuccessful induction of labor (each 1.9%).

### 3.5. Perinatal Fetal Outcome

Singleton mothers delivered at a median of 40 GW and twin mothers gave birth 2 weeks earlier (Table 4)—a difference which persisted after adjustment, resulting in gestational age at delivery being on average 2.63 weeks lower in twin pregnancies (95% CI: −3.11; −2.14) (Figure 1). Preterm birth before 37 GW was more common in twin pregnancies (26.0% vs. 4.6%). Similarly, prematurity <34 GW (5.5% vs. 1.3%) and <28 GW (1.4% vs. 0.2%) was more frequent in twin pregnancies (Table 4). Also, after adjustment, the odds of preterm birth were substantially higher for twin pregnancies (<37 GW: OR = 6.59, 95% CI: 3.38; 12.29, and <34 GW: OR = 4.84, 95%CI:1.35; 13.60) (Figure 2).

Median umbilical cord pH levels were 7.25 in singletons and 7.27 in twins, with corresponding median BE values of −4.00 and −3.20 mmol/L, respectively. Acidosis, defined as an umbilical cord pH < 7.2, was more frequently observed in singleton newborns (23.9% vs. 13.5%), just as severe acidosis with a pH < 7.1 (2.4% vs. 1.4%) (Table 4). After adjusting for confounding, BE was 1.38 mmol/L higher (95% CI: 0.62; 2.14) and umbilical cord pH 0.03 units higher (95% CI: 0.01; 0.04) in twins compared to singletons (Figure 1). When acidosis is defined with a pH < 7.2 we see the same trend with lower odds for acidosis in twins (OR = 0.51, 0.22–1.04), but not with a cut-off for acidosis defined as pH < 7.1 (OR = 1.37; 95% CI: 0.22; 4.71) (Figure 2).

**Figure 3:**
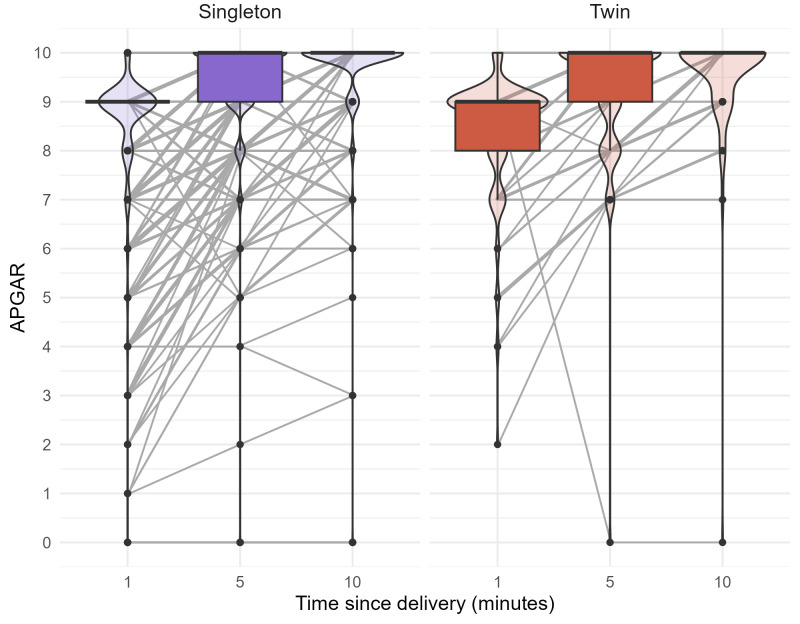
APGAR scores over time. APGAR scores at 1, 5, and 10 min after delivery displayed using boxplots and violin plots, stratified by singletons and twins. Median APGAR scores are identical in both groups (9/10/10). However, twins displayed a tendency towards lower individual scores, especially 1 min after delivery (median (IQR): 9 (8, 9) vs. 9 (9, 9)).

The proportion of newborns with APGAR scores of 7 or higher was comparable between singletons and twins at 1 min (94.7% vs. 93.8%), 5 min (98.5% vs. 99.3%), and 10 min (99.2% vs. 99.3%) after delivery (Table 4). While the median scores at 1, 5, and 10 min after delivery were identical between both groups (9/10/10), twins exhibited a greater variability, with a higher tendency towards lower individual scores. At 1 min after delivery, the median and IQR were 9 (9, 9) in singletons and 9 (8, 9) in twins (Figure 3). Taking confounders into account, the odds of an APGAR score <7 at 1 min were higher for twins (OR = 1.61; 95% CI: 0.54; 3.82) (Figure 2).

Shoulder dystocia was observed in twelve women in the singleton group (0.7%), while none occurred in the twin group. There were six IUFDs in the singleton group (0.4%) and none in the twin group (Table 4).

Differences in birthweight and birthweight percentiles were noted between the groups. The median birthweight was 3450 g in singletons and 2665 g in twins. Correspondingly, the median birthweight percentile was 57 for singletons and 30 for twins (Table 4). Taking potential confounders into account, twins still weighed on average 0.83 kg less than singletons (95% CI: −0.98; −0.69) and correspondingly scored 17.3 points lower in terms of birthweight percentiles (95% CI: −23.5; −11.1]) (Figure 1).

The proportion of IUGR was higher in twin pregnancies (3.6% vs. 1.9%), as was the proportion of SGA (12.9% vs. 8.4%). A fetal weight below the 30th percentile was nearly twice as common in twins (49.3% vs. 25.4%) (Table 4). Adjusting confounding, twins still had 6-fold the odds of a fetal birthweight < 30th percentile (OR = 6.11; 95% CI: 3.49; 11.12), 2.5-fold the odds of SGA (OR = 2.50; 95% CI: 1.23; 4.76), and 3-fold the odds of IUGR (OR = 3.37; 95% CI: 0.96; 9.11) (Figure 2). Conversely, we observed that 16.1% of singletons were LGA, while none of the twins were (Table 4).

## 4. Discussion

### 4.1. Main Findings

This study highlights several key differences between singleton and twin pregnancies affected by GDM. Women with twin pregnancies were of slightly advanced maternal age with fewer previous pregnancies and births, received the 75 g OGTT earlier in pregnancy and were less likely to require insulin therapy. The glucose levels in the 75 g OGTT were similar between twin and singleton mothers.

Vaginal deliveries were more than twice as common in the singleton group, while CS, especially primary CS, was substantially more frequent in the twin group, but the proportion of emergency cesareans was comparable between both groups. Twin mothers delivered significantly earlier than singleton mothers. Regarding neonatal outcomes, the odds of acidosis with a pH < 7.2 were lower in twins, while comparable odds of acidosis with a pH < 7.1 were observed. The proportions of well-adapted newborns were comparable between the groups at all times, with higher odds of an APGAR score < 7 at 1 min in twins. Twins also had increased odds of SGA, IUGR, and growth < 30th percentile, while LGA newborns were observed only in the singleton group.

### 4.2. Baseline Characteristics

In recent decades, maternal age at first pregnancy increased, driven by social changes as well as improved accessibility and advances in reproductive medicine [29]. Despite slightly higher maternal age in our twin group, over half of singleton mothers in our cohort were in their third or later pregnancy, whereas two thirds of twin mothers were in their first or second. Additionally, more than 50% of the twin mothers were nulliparous. Although ART-associated multiple pregnancies have declined, twin pregnancies remain much more common after ART than spontaneous conception as of 2019 [30]. Unfortunately, our data lacked the mode of conception, limiting interpretation of whether the higher proportion of nulliparity among women with twin pregnancies reflects an association with ART.

### 4.3. Diagnostics and Metabolism

In our study, women with twin pregnancies underwent the 75 g OGTT earlier than women with singleton pregnancies, leading to earlier referral to the GDM consultation. Moreover, in our study, the 75 g OGTT was more frequently performed as a first-line screening measure in women with twin pregnancies. This suggests that twin pregnancies were more often associated with GDM risk factors, prompting earlier diagnostic evaluation. Interestingly, this was not explained by a higher BMI among twin mothers, who instead tended to have a lower BMI. This discrepancy may be attributed to advanced maternal age or other unmeasured factors, such as GDM in a previous pregnancy or family history of diabetes mellitus [9]. Although absolute as well as relative gestational weight gain was, as expected, slightly higher in twin mothers, it did not substantially differ from the weight gain of singleton mothers. Another possibility might be earlier assessment due to the presence of a twin pregnancy, which several studies have identified as an independent risk factor for GDM [13,14,15]. Despite differences in timing and screening approach, glucose levels in the 75 g OGTT were similar between groups, while previous research has reported lower or less frequently elevated fasting and higher or more frequently elevated postprandial glucose levels in twin mothers [21,31].

Following a GDM diagnosis, women are advised to implement certain lifestyle changes, including a healthy diet and regular physical activity, alongside routine blood glucose monitoring. If dietary treatment alone fails to achieve satisfactory glycemic control, insulin therapy is required. In Germany, insulin therapy is initiated according to strict guideline-based algorithms provided by the AWMF. Current clinical guidelines do not differentiate between singleton and twin pregnancies in terms of diagnostic thresholds and glycemic target values [1]. In our study cohort, twin mothers were less likely to require insulin than singleton mothers, aligning with previous findings [21,32,33], although another study reported similar proportions of insulin therapy between the groups [31]. Catic et al. have recently identified higher maternal BMI, among others, to be a predictor for insulin requirement [34], and the lower BMI observed in our twin group may partly explain their lower need for insulin therapy. Additional physiological factors characteristic of twin pregnancies may influence glucose metabolism. Levels of lactogenic placental hormones, such as human placental lactogen (hPL), rise during pregnancy to ensure sufficient glucose supply to the fetus and are linked to increasing insulin resistance, while—in healthy pregnancies—possibly increasing insulin secretion at the same time. Since hPL levels correlate with placental mass, which is increased in twin pregnancies [35,36], this may affect glucose regulation. On the other hand, twin mothers tend to have higher basal metabolic rates than singleton mothers [37], and the overall fetal glucose demand might be higher with two fetuses, possibly reducing the need for exogenous insulin by increased glucose uptake. Beyond metabolic differences, clinical decision-making when managing twin pregnancies may also be influenced by the elevated risk of fetal growth restriction, which is particularly emphasized in the German AWMF guideline [20]. This may lead to more cautious initiation of insulin therapy, even if glycemic target values defined in the GDM guideline provided by the AWMF are exceeded.

### 4.4. Maternal Outcome

The slightly higher rate of preeclampsia among twin mothers in our cohort likely reflects the generally increased risk of preeclampsia in twin pregnancies [17]. The odds were comparable for twin and singleton mothers after adjusting for confounding. However, a higher risk of preeclampsia in twin compared to singleton pregnancies affected by GDM was previously reported [21,33,38]. The role of GDM in twin pregnancies on the incidence of hypertensive disorders of pregnancy (HDP) remains unclear, with some studies suggesting comparable risks independent of GDM status [21,39] and some indicating increased risk in pregnancies with GDM [38,40,41]. Liu et al. found GDM to be a risk factor for HDP only in monochorionic pregnancies [42].

Twin mothers in our cohort were substantially more likely to deliver via CS, and especially primary CS, while the odds for emergency CS and operative vaginal birth were comparable between twin and singleton mothers. Previous studies have reported increased percentages of CS of up to 80% or more in twin pregnancies with GDM [21,43]. However, Ronco et al. found no statistically significant increased odds of CS in twin mothers with GDM compared to a control group of twin mothers without GDM (82.8% vs. 77%; OR = 1.2; 95% CI: 0.9–1.8; *p* = 0.104), suggesting no significant impact of GDM on birth mode in twin pregnancies [21]. Moreover, the proportion of CS in our group of twin mothers with GDM is similar to the proportion of CS in all multiple births in Germany in 2021, suggesting no relevant impact of GDM on birth mode in twin pregnancies [7]. A large proportion of CSs in our twin group was due to fetal position, previous CS, and fetal growth anomalies including IUGR and growth discrepancies, but not LGA. A total of 15% of women were scheduled to have a primary CS but received a CS after onset of labor, and in another 19% the indication for CS was not specified, which could be accounted for by factors such as patient or physician preference.

### 4.5. Fetal Outcome

Greco et al. observed that in singleton and twin pregnancies complicated by GDM, the risk of preterm birth was significantly increased compared to those without GDM [23]. As expected, twin mothers in our study delivered earlier on average, with a higher proportion of preterm births. This aligns with a meta-analysis by Tu et al., which reported that singleton mothers with GDM had a significantly lower risk of preterm birth compared to twin mothers with GDM (OR = 0.07; 95% CI: 0.06–0.09; *p* < 0.001) [43]. Twin pregnancies inherently carry a higher baseline risk of obstetric complications [44,45], and the combination of increased physical strain on the uterus, higher risk of complications, and the necessity for early interventions in many twin pregnancies makes them significantly more likely to result in preterm delivery compared to singleton pregnancies [46]. A study by Sheehan et al. reported 26-fold odds of preterm birth in twin pregnancies (OR = 26.12; 95% CI: 19.77–34.51; *p* <0.001), while GDM showed a less pronounced association with preterm birth in a univariate analysis (OR = 1.55; 95% CI: 1.29–1.84; *p* <0.001) [47]. These results are consistent with a meta-analysis that found no difference in gestational age at delivery in GDM versus non-GDM twins [48].

In singleton pregnancies, GDM is a well-established risk factor for the development of an LGA fetus [2]. Although several studies have also reported a higher risk of LGA in twin pregnancies complicated by GDM [23,38,49,50], twins appear to be less frequently affected by LGA than singletons in the context of GDM [21,50], possibly leading to a decreased rate of complications such as shoulder dystocia in these pregnancies as well. Consistent with these findings, we did not identify any cases of LGA or shoulder dystocia in twins. In contrast, we found higher odds of IUGR and SGA in twins, consistent with previous research [43]. The NICHD Fetal Growth Studies further support this, showing that by 35 weeks, nearly 40% of twins would be considered SGA by singleton growth standards [51]. A higher risk of IUGR and SGA in twin versus singleton pregnancies, independent of GDM status, was also reported in a large study by Esteves-Pereira et al. [19]. These findings indicate that the increased likelihood of growth restriction we observed in our twin cohort likely reflects inherent characteristics of twin pregnancy rather than being mediated by GDM. Indeed, previous research suggests that the accelerated growth induced by GDM possibly provides a metabolic advantage in twin pregnancies and might even be a protective factor against certain adverse outcomes, including SGA, low APGAR scores and perinatal mortality [23,41,50]. Since low birthweight is one of the main risk factors for neonatal morbidity in twins, this growth-promoting effect of GDM could help mitigate some of their inherent vulnerability [23]. Furthermore, Fox et al. reported an association between improved glycemic control and SGA in twin pregnancies, raising the question of whether target glucose levels should be more permissive in twin pregnancies [52]. Due to the absence of a healthy control group in our study, we were unable to further evaluate the possible protective role of GDM based on our data.

The similar OGTT values we observed in twin and singleton pregnancies along with the much lower insulin requirements and absence of LGA in twin pregnancies raise the important question of whether the current guideline approach is suitable for twin gestations. Women with twin pregnancies, while showing glycemic levels comparable to those with singleton pregnancies at the time of diagnosis, seem to experience a milder course of GDM. Existing guidelines do not address the unique physiological adaptations distinguishing multiple from singleton pregnancies, despite their potential impact on GDM severity. This aspect may potentially contribute to an overdiagnosis of GDM in twin pregnancies. However, making specific recommendations regarding distinct diagnostic thresholds would go beyond the scope of our data, and prospective studies are required to further investigate this issue.

Postnatal adaptation, measured by APGAR scores, was broadly similar between twins and singletons, though twins displayed a tendency towards slightly lower individual scores and higher risk of a low 1 min APGAR. Since twin mothers delivered earlier and the risk of low Apgar scores increases with decreasing gestational age [53], this could explain the lower scores in twin births. However, Greco et al. reported no increased risk of low Apgar scores in GDM versus non-GDM pregnancies, in either twins or singletons [23].

Six IUFDs (0.4%) were observed in our singleton group and none in the twin group. Intensified prenatal monitoring in GDM pregnancies and a lower threshold for delivery, especially in twin pregnancies, might contribute to such low numbers of IUFD in both groups. Umbilical cord blood analyses showed more favorable pH and base excess (BE) values in twins, with lower odds of mild acidosis (pH < 7.2) in twins. This may be attributed to the higher frequency of CS in twin pregnancies, which may reduce exposure to intrapartum stress associated with vaginal birth [54]. Despite these findings, the frequencies of severe acidosis were comparable between the two groups, suggesting that while CS may mitigate mild acid–base disturbances, it does not impact the risk of more severe metabolic compromise.

### 4.6. Limitations

A key limitation of this study is its retrospective design, resulting in missing data and potential reporting bias, as the documentation’s primary aim was clinical GDM management. Additionally, our data were derived from a single large tertiary perinatal center and may therefore not be representative of other populations. Potentially, patients perceived by their primary physicians to have higher perinatal risk or more severe GDM were more likely to be referred to our clinic, introducing selection bias. Moreover, the absence of control groups, specifically a non-GDM twin group, limits the ability to distinguish whether differences in outcomes are linked to GDM itself or merely reflect baseline risks inherent to twin pregnancy. Another relevant limitation is the lack of information on the mode of conception in our data. Given that ART is frequently used in Germany and strongly associated with both twin pregnancy and metabolic risk, it could represent a strong confounding factor [30,55]. We also could not report patient compliance and glycemic control throughout the pregnancies.

## 5. Conclusions

Our findings support distinct risk profiles for GDM in twin and singleton pregnancies. Despite similar glycemic profiles in the 75 g OGTT, only a small proportion of twin mothers require insulin therapy, suggesting a unique physiological adaptation of maternal glucose metabolism to a pregnancy with two fetuses. Common complications in twin pregnancies, such as reduced growth, preterm birth, and CS, seem to be inherent characteristics of multiple gestations rather than effects of GDM. In contrast, adverse outcomes that commonly affect singleton pregnancies, such as LGA and insulin dependency, are rarely observed in twin pregnancies. These findings highlight the importance of individualized GDM management strategies tailored to the specific dynamics of twin and singleton gestations. Prospective studies are needed to further elucidate the implications of GDM in twin pregnancies.

## Figures and Tables

**Table 1:** Maternal baseline characteristics, stratified by singleton vs. twin pregnancies, as recorded at time of initial presentation at the GDM consultation clinic.

	Singleton (n = 1664)		Twin (n = 73)	
	Median (IQR)/n (%)	n	Median (IQR)/n (%)	n
GW at first presentation	30 (28, 34)	1635	28 (27, 30)	69
Maternal age in years	33 (28, 36)	1663	34 (32, 36)	73
BMI (kg/m^2^)	27.5 (23.9, 32)	1617	25.75 (23.55, 29.95)	60
Gravidity	3 (2, 4)	1662	2 (1, 3)	71
Gravidity				
*1*	365 (22.0%)	1662	22 (31.0%)	71
*2*	421 (25.3%)		25 (35.2%)	
*≥3*	876 (52.7%)		24 (33.8%)	
Parity	1 (0, 2)	1662	0 (0, 2)	71
Nulliparity	526 (31.6%)	1662	36 (50.7%)	71
Parity				
*1*	481 (28.9%)	1662	15 (21.1%)	71
*2*	323 (19.4%)		13 (18.3%)	
*≥3*	332 (20.0%)		7 (9.9%)	

IQR = interquartile range; GW = gestational week; BMI = Body Mass Index.

**Table 2:** Metabolism. The 50 g OGCT and 75 g OGTT diagnostic test results in singleton and twin mothers.

	Singleton (n = 1664)		Twin (n = 73)	
	Median (IQR)/n (%)	n	Median (IQR)/n (%)	n
50 g OGCT	905 (54.4%)	1664	26 (36.1%)	72
GW at 50 g OGCT	26 (25, 27)	890	25 (24.75, 26)	28
Blood glucose 50 g OGCT (mg/dL)	154 (141, 171)	863	161.5 (143, 177)	26
Pathological 50 g OGCT	791 (91.6%)	864	25 (96.2%)	26
GW at 75 g OGTT	27 (25, 28)	1624	26 (25, 28)	68
75 g OGTT before GW 24 + 0	185 (11.4%)	1624	10 (14.7%)	68
Fasting blood glucose	96 (91, 103)	1664	95 (90, 101)	73
1 h blood glucose	183 (159, 203)	1663	183 (165.25, 198)	70
2 h blood glucose	139 (117, 161)	1664	144 (119.25, 169)	70

IQR = interquartile range; OGCT = oral glucose challenge test; GW = gestational week; OGTT = oral glucose tolerance test.

**Table 3:** Maternal outcome and birth mode, stratified by singleton vs. twin pregnancies.

	Singleton (n = 1664)		Twin (n = 73)	
	Median (IQR)/n (%)	n	Median (IQR)/n (%)	n
Weight gain in kg	11 (7, 15)	1381	12.4 (7.4, 16.4)	51
Relative weight gain in %	14.9 (8.8, 22.3)	1381	17.3 (10.2, 23.1)	51
Insulin therapy	371 (22.3%)	1664	4 (5.5%)	73
Insulin type		1664		73
*-Long-acting insulin only*	229 (13.8%)		1 (1.4%)	
*-Short-acting insulin only*	26 (1.6%)		0 (0%)	
*-Combined insulin*	116 (7%)		3 (4.1%)	
Vaginal birth	959 (57.7%)	1663	36 (24.6%)	73
Vaginal spontaneous birth	834 (50.2%)	1663	26 (17.8%)	73
Vaginal operative birth	125 (7.5%)	1663	10 (6.8%)	73
Cesarean section	704 (42.3%)	1663	110 (75.3%)	73
Primary cesarean section	362 (21.8%)	1663	78 (53.4%)	73
Emergency cesarean section	342 (20.6%)	1663	32 (21.9%)	73
Preeclampsia	55 (3.3%)	1664	4 (5.6%)	73

IQR = interquartile range.

**Table 4:** Fetal outcomes, stratified by singleton vs. twin pregnancies.

	Singleton (n = 1664)		Twin (n = 73/146)	
	Median (IQR)/n (%)	n	Median (IQR)/n (%)	n
GW at delivery	40 (39, 41)	1663	38 (36, 38)	73
Prematurity *<37 GW*	76 (4.6%)	1663	19 (26.0%)	73
*<34 GW*	22 (1.3%)		4 (5.5%)	
*<28 GW*	3 (0.2%)		1 (1.4%)	
Umbilical cord pH	7.25 (7.20, 7.29)	1642	7.27 (7.24, 7.30)	141
Base Excess	−4.00 (−6.20, −2.20)	1625	−3.20 (−5.05, −1.70)	138
Acidosis pH < 7.2	392 (23.9%)	1642	19 (13.5%)	141
Acidosis pH < 7.1	39 (2.4%)	1642	2 (1.4%)	141
APGAR ≥ 7 at 1 min	1560 (94.7%)	1647	135 (93.8%)	144
APGAR ≥7 at 5 min	1623 (98.5%)	1647	143 (99.3%)	144
APGAR ≥7 at 10 min	1634 (99.2%)	1647	143 (99.3%)	144
Shoulder dystocia	12 (0.7%)	1664	0 (0%)	146
IUFD	6 (0.4%)	1664	0 (0%)	146
Birthweight in g	3450 (3105, 3792.5)	1663	2665 (2386.25, 2886.25)	146
Birthweight percentile	57 (29, 81.25)	1660	30 (17, 51)	140
IUGR	31 (1.9%)	1660	5 (3.6%)	140
SGA	139 (8.4%)	1660	18 (12.9%)	140
Fetal weight <30th p.	422 (25.4%)	1660	69 (49.3%)	140
LGA	268 (16.1%)	1660	0 (0%)	140

IQR = interquartile range; GW = gestational week; IUFD = intrauterine fetal death; IUGR = intrauterine growth restriction (defined as growth <3rd percentile); SGA = small for gestational age (defined as growth <10th percentile); LGA = large for gestational age (defined as growth >90th percentile).

## Data Availability

The datasets generated and analyzed during the current study are available from the corresponding author upon reasonable request.
